# Equivariant diffusion for structure-based de novo ligand generation with latent-conditioning

**DOI:** 10.1186/s13321-025-01028-x

**Published:** 2025-05-31

**Authors:** Tuan Le, Julian Cremer, Djork-Arné Clevert, Kristof T. Schütt

**Affiliations:** Machine Learning Research, Pfizer, Friedrichstraße 110, Berlin, 10117 Germany

**Keywords:** Structure-based drug discovery, Machine learning, Deep learning, Diffusion, Latent diffusion, Graph neural network, Equivariance

## Abstract

We introduce PoLiGenX, a novel generative model for *de novo* ligand design that employs latent-conditioned, target-aware equivariant diffusion. Our approach leverages the conditioning of the ligand generation process on reference molecules located within a specific protein pocket. By doing so, PoLiGenX generates shape-similar ligands that are adapted to the target pocket, enabling effective applications in target-aware hit expansion and hit optimization. Our experimental results underscore the efficacy of PoLiGenX in advancing ligand design. Notably, docking analyses reveal that the ligands generated by PoLiGenX show enhanced binding affinities relative to their reference molecules, all while retaining a similar molecular shape, but  also retaining better poses with lower strain energies and less steric clashes. Furthermore, the model promotes substantial chemical diversity, facilitating the exploration of broader and more varied chemical spaces. Importantly, the generated ligands were assessed for drug-likeness using Lipinski’s rule of five, demonstrating superior adherence to drug-likeness criteria compared to the reference dataset. This work represents a step forward in the controlled and precise generation of therapeutically relevant *de novo* ligands tailored for specific protein targets, contributing to progress in computational drug discovery and ligand design.

## Scientific Contribution

We present a latent-conditioning method within diffusion models to enable the controllable generation of ligands in structure-based drug design that are similar to a reference ligand. We show that the generated ligands obtained via latent-conditioning achieve favorable ligand poses with reduced steric clashes and lower strain energies compared to diffusion models that only condition on the protein pocket alone. We demonstrate that the ligand generation can be further constrained using an importance sampling algorithm with external surrogate models that account for molecular properties such as synthetic accessibility.

## Introduction

**Fig. 1:**
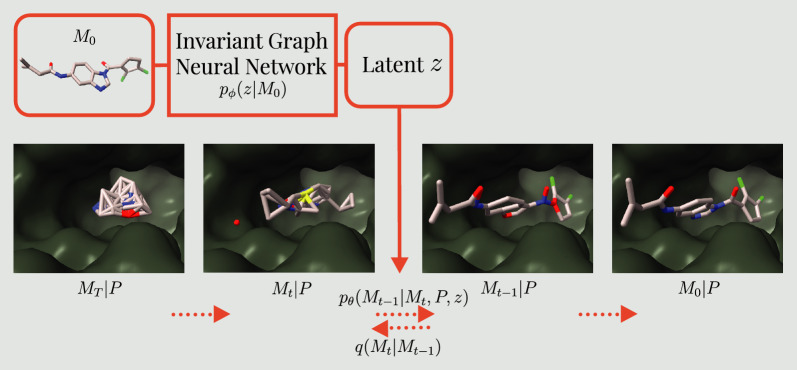
Graphical model of the proposed latent diffusion model. The encoded ligand *z* serves as input to the diffusion model $p_\theta$ to steer the generation process of new ligands $M_0$

In recent years, the intersection of artificial intelligence (AI) and drug discovery has shown potential to transform the approaches to identify novel therapeutic compounds. AI-enabled structure-based drug discovery has emerged as a promising research avenue, in particular using equivariant target-aware diffusion models. By conditioning the diffusion process on a receptor, these models have demonstrated the capacity to generate *de novo* ligands with enhanced affinity [1–5]. However, failing to consider the essential chemical properties for target binding can lead to a significant lack of specificity and result in ineffective drug candidates. Moreover, these candidates must exhibit favorable absorption, distribution, metabolism, excretion (ADME), and toxicity profiles. Designing ligands from scratch without addressing these critical properties may produce molecules with poor bio-availability or potential toxicity, thereby limiting their therapeutic potential. This challenge is further exacerbated by the often sparse and noisy data available for developing effective generative deep learning models. However, there is a considerable promise during the hit expansion phase of drug discovery. This crucial stage involves enhancing and exploring the chemical space around promising hits that are already identified through high-throughput screening or other well-proven methods. In this study, we introduce PoLiGenX (**Po**cket-based **Li**gand **Gen**erator for hit e**X**pansion) that generates ligands *de novo* within a protein binding pocket. However, PoLiGenX is given a seed molecule, such as a hit candidate or an initial scaffold, guiding the generation process to improve its efficacy. We enhance the capabilities of the existing equivariant diffusion model EQGAT-diff [3] by conditioning a latent encoding of the seed. It is learned by an invariant graph neural network that is jointly trained with the generator. The setup ensures that the newly generated ligands retain structural characteristics of the seed molecules while undergoing necessary chemical modifications and diversification. Our proposed approach adds a new level of control to the process of generating *de novo* ligands, aligning it more closely with the specific needs of targeted drug design, particularly during the hit expansion phase, while not restricting it too much for effective chemical space exploration. A graphical overview is presented in Fig. 1.

## Related work

Deep generative modeling in the life sciences has emerged as a promising research domain, offering innovative approaches to molecular design. Recent studies, such as those by Xu et al. [6], Jing et al. [7], have utilized Denoising Diffusion Probabilistic Models (DDPMs) [8–11] to predict the 3D coordinates of molecules, leveraging the capabilities of 3D equivariant graph neural networks. In the *de novo* molecular design setting, another line of research focuses on generating atomic coordinates and elements directly, employing autoregressive models [12–14]. Hoogeboom et al. [15] introduced the E(3) Equivariant Diffusion Model (EDM) for *de novo* molecule design. It simultaneously learns atomic elements and coordinates while treating chemical elements as continuous variables, enabling the application of DDPM formalism. Building on EDM, subsequent works have extended diffusion models to specific tasks, including linker design [16] and structure-based ligand modeling [2, 3, 5]. In the realm of shape-conditioned molecule generation, Adams and Coley [17] (SQUID) and Chen et al. [18] (ShapeMol) recently proposed incorporating the shape of a seed molecule into the generation process. Both approaches use an equivariant surface encoding of the seed molecule, with SQUID employing variational auto-encoding on molecular graphs, focusing on fragment-based design. ShapeMol adapts SQUID to the 3D space by integrating an equivariant diffusion model. However, neither approach incorporates protein receptor conditions into the generation process. To address this limitation, we propose a simplified yet effective approach that employs reference molecules represented in a latent representation as in Cremer et al. [19] and detailed in the following sections. We extend their analysis for the latent-conditioned generated ligands by evaluating generated poses with respect to steric clashes to the protein pocket, the ligand strain energies, as well as the recovery of known hydrogen bonds.

## Methods

We investigate the generation of molecular structures *M* in a *de novo* setting conditioned on a protein pocket *P*, i.e., building a generative model $p_\theta (M|P)$. We achieve this by extending EQGAT-diff [3]. In our setup, a noisy ligand $M_t = (X_t, H_t, E_t)$ - representing perturbed atomic coordinates, element types, and bond features - is used, and the diffusion model predicts the uncorrupted data modalities. Specifically, for continuous coordinates, the reverse distribution is parameterized by a multivariate Gaussian, while discrete-valued modalities follow a categorical distribution. We refer to Le et al. [3] for further details. While models like EQGAT-diff, TargetDiff, or DiffSBDD generate ligands in the context of a protein pocket, they do not constrain the generated ligands to preserve properties like shape or chemical similarity during training. In contrast, we include a latent variable $z \in \mathbb {R}^k$ that relates to the input molecule. The latent *z* may serve as a shape conditioning that also comprises chemical information like the atom composition of the molecule. The graph encoder $q_\phi : \mathcal {X}^M \rightarrow \mathbb {R}^k$ is invariant to permutation, rotation, and translation of atoms [20, 21]. Following Adams and Coley [17], chemical similarity of two molecules is measured as the Tanimoto similarity of ECFP4 fingerprints (2048 bits) computed by the RDKit, while shape similarity is defined by Gaussian descriptions of molecular shape in the form of atom-centered Gaussians and calculated by the volume overlaps between them, as in Adams and Coley [17].

To model the dependence on the latent variable *z*, we include a variational distribution $q_\phi (z|M_0)$ similar to Luo and Hu [22], Zeng et al. [23] and obtain the evidence lower bound (ELBO) as

1 $$\begin{aligned} \begin{aligned} p_\theta (M_0|P) = \mathbb {E}_{q(M_{1:T}|M_0) q_\phi (z|M_0)}[\frac{p_\theta (M_0, M_{1:T}, z|P)}{q(M_{1:T}|M_0) q_\phi (z|M_0)}]&\ge \\ \mathbb {E}_{q(M_{1:T}|M_0) q_\phi (z|M_0)}[\log \frac{p_\theta (M_0, M_{1:T}, z|P)}{q(M_{1:T}|M_0) q_\phi (z|M_0)}]&=\\ \mathbb {E}_{q(M_{1}|M_0) q_\phi (z|M_0)}[\log p_\theta (M_0|M_1, P, z)]&+\\ \mathbb {E}_{q(M_T|M_0)q_\phi (z|M_0)}[\log \frac{p(M_T|z)}{q(M_T|M_0)}]&-\\ D_{KL}({q_\phi (z|M_0)} || p(z)) - \sum _{t=2}^T \mathbb {E}_{q(M_t|M_0)q_\phi (z|M_0)}[L_{t-1}], \end{aligned} \end{aligned}$$

where the diffusion loss $L_{t-1}$ is per time-step and defined as $L_{t-1} = D_{KL}(q(M_{t-1} | M_t, M_0) || p_\theta (M_{t-1}|M_t, P, z))$. We refer to Appendix A for the ELBO derivation in the case of Gaussian diffusion, which is an extended contribution to Cremer et al. [19].

We extend the diffusion model by conditioning on *z* and train $p_\theta (M|P, z)$ to minimize the KL divergence to the tractable reverse distribution, which is achieved when predicting the original data points $\hat{M}_0$ [24, 8, 3]. Similar to prior works, we optimize the diffusion loss $L_{t-1}$ by drawing steps per mini-batch instead of the entire trajectory. We adopt a Gaussian prior for the latent distribution, i.e., $p(z) \sim N(0, I)$, and enforce a smooth latent space by choosing the maximum mean discrepancy (MMD) loss [25] over the KL divergence. The prior distribution for the ambient data space, i.e., $M_T$, is a 0-Center-of-Mass (CoM) Gaussian for coordinates and an empirical categorical distribution for discrete data types from the training set as discussed in Le et al. [3]. During training, we sample a batch of pocket-ligand pairs and a step $t\in \{1, \dots , 500\}$. Next, we encode the ligands $M_0$ into latents *z*, apply the noise process to the ligands to obtain $M_t$, and minimize the diffusion loss while providing *z* as an additional input via adaptive layer normalization [26] (see Eq. (2)) alongside the protein pocket *P*.

**Fig. 2:**
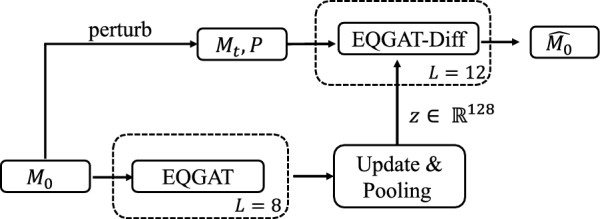
High level overview of the model computation. The lower part describes the group invariant graph encoder that inputs the ground-truth ligand $M_0$ and outputs the latent embedding *z*. EQGAT-diff inputs the perturbed ligand $M_t$ next to the pocket as well as latent representation as context. EQGAT-diff is tasked in predicting the uncorrupted ligand $M_0$

We leverage the EQGAT-diff architecture as proposed in Le et al. [3] and modify it to process the pocket-ligand (PL) complex as in [1]. To build the PL complex, we use the creation strategy from DiffSBDD [5] with a 5 Ångström cutoff. In summary, a PL complex is built by iterating over the atoms in each residues in a protein and computing all pairwise distances to all ligand atoms. If any distance between the atom of the residue to any ligand atom is below the cutoff, the entire residue is included in the pocket.

To form the PL representation for message-passing we stack ligand and pocket features. Ligands represented as $(X_l, H_l, E_l)$, indicating spatial coordinates, atom and edge types are in same fashion generated as in EQGAT-diff. We create a fully-connected graph and include the bond adjacency with features (single, double, triple and no bond) into the edge features.

The edge features in the pocket representation $(X_p, H_p, E_p)$, are all set to the no bond type. The edge indices and connectivity for pocket-pocket and ligand-pocket/pocket-ligand interaction are obtained through a radius graph with 5Å cutoff.

An important aspect for training diffusion models is the noise scheduler, particularly when different modalities such as coordinates, atom- and bond-types are learned. In similar fashion to the work by Vignac et al. [27], we leverage an adaptive noise scheduler

$$\bar{\alpha }^{t}=\cos \left( \frac{\pi }{2} \frac{(t / T+s)^{\nu }}{1+s}\right) ^{2},$$

with $\nu _{r}=2.5, \nu _{y}=1.5, \nu _{x}=1.0$ denoting for atom coordinates, bond types, atom types, respectively. The rationale behind these coefficients is to enable for slower decay of the signals of coordinates, bond- and atom-types.

We provide a high-level computational workflow in Fig. 2.

### *G*-invariant latent encoder EQGAT

The group-invariant graph encoder is implemented using the EQGAT message passing layer as proposed by Le et al. [20]. After $L=8$ round of message passing, we extract the SO(3)-invariant scalar node embeddings $H \in \mathbb {R}^{N\times k}$ and leverage a gated equivariant transformation as proposed in the original EQGAT architecture followed by a SoftmaxAttention-pooling [28] along the node embeddings, to achieve the SO(3) as well as permutation invariant latent embedding $z \in \mathbb {R}^k$. We set the latent dimension to $k=128$.

The derivation of the ELBO requires the variational distribution $z \sim q(z|x_0)$. Such distribution can be simply obtained through, e.g., reparameterization using Gaussian variables as $z=\mu _\phi (x_0) + \sigma _\phi (x_0)\odot \epsilon ,~~\epsilon \sim N(0, I_k)$. Here, we follow the approach of deterministic (V)AE where the standard deviation converges towards 0, i.e. each latent being a dirac-delta point mass in $\mathbb {R}^{k}$.

### Incorporating the latent *z* into EQGAT-diff

We include the latent embedding $z\in \mathbb {R}^k$ derived from the group-invariant graph encoder, via adaptive layer normalization (AdaLN) to fuse the global latent embedding with the node embeddings $S\in \mathbb {R}^{N\times k}$ from the current point cloud that EQGAT-diff performs after every message-passing layer. That is, instead of having shared learnable affine parameters for every input, we compute the affine parameters based on the latent (style) *z*, which is inspired by adaptive instance normalization introduced by Huang and Belongie [26].

2 $$\begin{aligned} \text {AdaptiveLN}(H, z, \lambda ) = \lambda s_\theta (z) \odot \left( \frac{H - \mu (H)}{\sigma (H)}\right) + \lambda b_\theta (z), \end{aligned}$$

where $\mu , \sigma$ are functions that compute the mean and standard deviation embeddings $\in \mathbb {R}^k$ from the hidden node embeddings $H\in \mathbb {R}^{N\times k}$ and $\lambda \in (0, 1]$. The influence of the latent embedding is enforced through the scale and shift operation obtained through the transformations $s_\theta , b_\theta$ which are both simple linear layers.

### Training

We train PoLiGenX under the data parameterization leveraging Gaussian diffusion for coordinates, and categorical diffusion for discrete-valued data modalities like chemical elements and bond types as outlined above. Therefore, the loss function for a sampled time-step on the diffusion loss reads

3 $$\begin{aligned} L_{t-1} = w_s(t) \Bigl ( \lambda _x || {X}_0 - \hat{{X}}_0||^2 + \lambda _h \text {CE}({H}_0, \hat{{H}}_0) + \lambda _e \text {CE}({E}_0, \hat{{E}}_0) \Bigr ), \end{aligned}$$

where CE refers to the cross-entropy loss and $(\lambda _x, \lambda _h, \lambda _e) = (3, 0.4, 2)$ are weighting coefficients adapted from [27]. The loss weighting $w_s(t)$ is modified truncated signal-to-noise ratio in Le et al. [3].

**Fig. 3:**
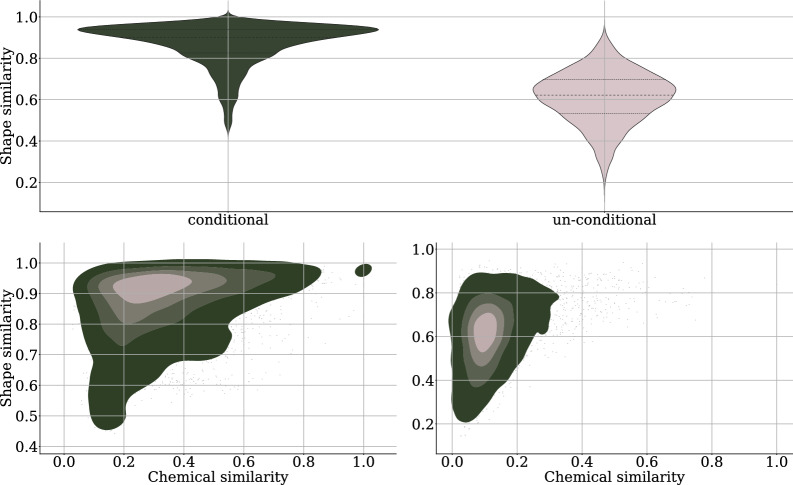
**Top**: Violin plot of the Tanimoto shape similarity evaluated across all test targets of the CrossDocked dataset. PoLiGenX (left) is compared to EQGAT-diff (right). In the conditional setting, the model generates significantly more shape-similar molecules. **Bottom**: Heatmap histogram comparing PoLiGenX (left) with EQGAT-diff with respect to Tanimoto shape and chemical similarity on the CrossDocked test set. The brighter the color, the higher the molecule count

The EQGAT-diff model uses 256 scalar and vector features each and 128 edge features across 12 layers of fully connected message passing. This corresponds to 13.4M trainable parameters.

The EQGAT graph-encoder model uses 128 scalar and vector features and 16 edge features across 8 layers of local message passing based on a 5Å radius cutoff. The graph-encoder has 2M trainable parameters. We compute the MMD loss between a batch of latents *Z* with a random batch drawn from an isotropic Gaussian to enforce a smooth latent space as proposed by Tolstikhin et al. [25]. Apart from that, we implement a simple (self)-supervised learning task in that the graph-encoder also predicts the number of nodes of the input molecule. This objective is optimized using cross-entropy loss, while additional self-supervised learning task might also be suitable, e.g., regressing on chemical properties that can be easily computed by the RDKit. Joint training of the diffusion and latent encoder model is crucial, as the latent variable guides thediffusion, and the encoder learns via the diffusion reconstruction loss in Eq. 3.

To train PoLiGenX, we use 8 NVIDIA A100 40GB GPUs with batch sizes of 8 each for 300 epochs on the CrossDocked2020 dataset. As optimizer we use AdamW with learning rate $2 \cdot 10^{-4}$, weight-decay of $1 \cdot 10^{-12}$, and gradient clipping for values higher than 10.

## Results

**Fig. 4:**
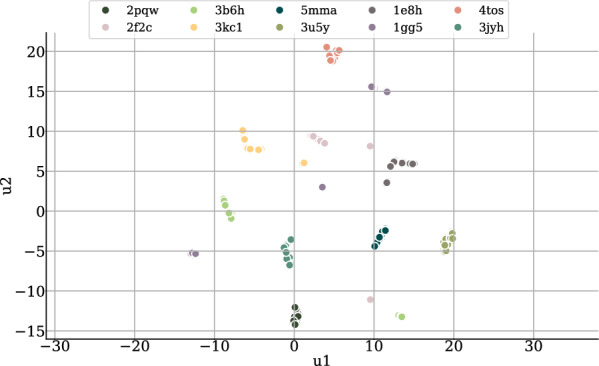
UMAP plot showing the 2D projections of the latent embeddings of 100 sampled ligands per target for ten randomly sampled test set targets

**Fig. 5:**
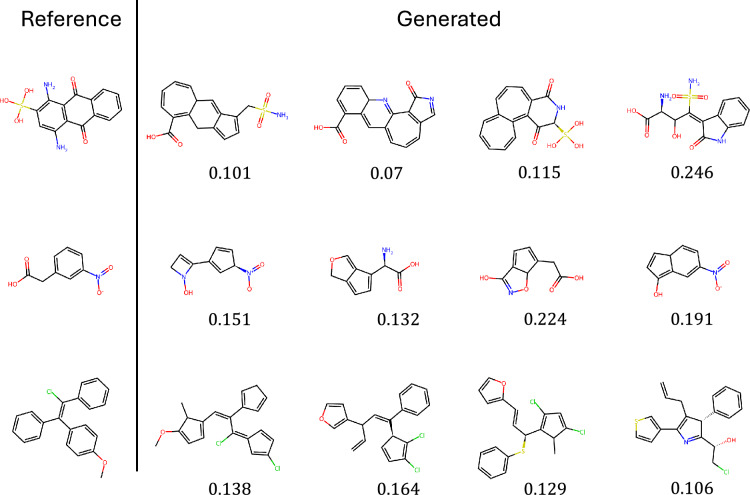
Reference molecules extracted from the CrossDocked2020 test split (left) and four generated molecules sampled randomly with PoLiGenX. Below each generated ligand we also show the chemical similarity to the reference ligand

We train PoLiGenX using the CrossDocked2020 dataset [29], following the dataset splits utilized in prior studies [2, 3, 30, 4, 5]. Unlike existing models, PoLiGenX conditions ligand generation not only on the protein pocket but also integrates a latent embedding of a ligand as an initial condition. This approach positions PoLiGenX as distinct, focusing on hit expansion by simultaneously enhancing specificity, chemical diversity, and binding affinity rather than solely operating as an unconditional *de novo* model. To assess the performance of PoLiGenX, we evaluate its ability to maintain the structural shape of a seed molecule while promoting chemical diversity. Figure 3 (top) illustrates the mean Tanimoto shape similarity on the CrossDocked test set for PoLiGenX (conditional) and EQGAT-diff (unconditional). The test set includes 100 ligand-pocket complexes, each sampled with 100 ligands, compared against reference ligands. PoLiGenX demonstrates significantly higher shape similarities across complexes. However, preserving shape similarity alone is insufficient for effective hit expansion. Figure 3 (bottom) shows the distribution of shape similarity against chemical similarity for conditional and unconditional sampling. PoLiGenX achieves a mean shape similarity of 0.87 and a chemical similarity of 0.33, compared to EQGAT-diff’s 0.64 and 0.12. This indicates that PoLiGenX not only preserves the structural shape but also facilitates the exploration of chemically diverse compounds. To evaluate the latent embedding’s expressiveness, Fig. 4 visualizes UMAP projections of latent embeddings for 100 ligands per receptor sampled from ten randomly chosen CrossDocked test set targets. The embeddings are effectively separated into distinct clusters, suggesting that PoLiGenX captures the contextual relationship between ligands and their corresponding protein receptors.

**Fig. 6:**
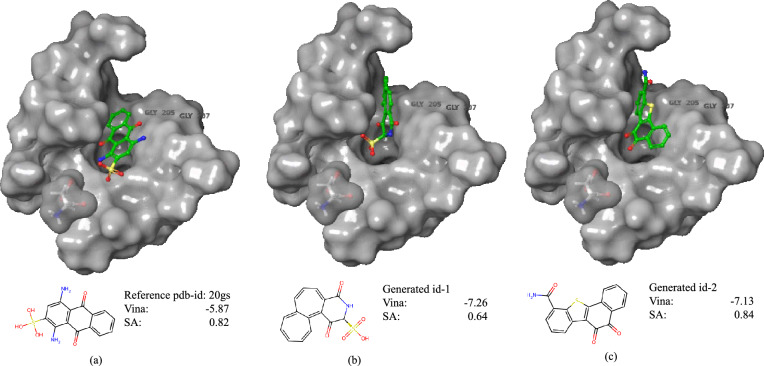
Ligands in the binding pocket of PDB id 20gs. Visualized are the reference ligand (**a**) and two generated samples from PoLiGenX (**b**-**c**). Generated ligand-1 is the sample with the lowest Vina score in the generated set, but maintains lower synthetic accessibility (SA) than the reference. The second generated sample with id-2 is superior to the reference ligand regarding Vina- and SA-score

**Table 1:** Docking performance on the CrossDocked2020 test set and ligands generated using PoLiGenX

Metric	Test set	PoLiGenX	PoLiGenX$^{\text {SA-guidance}}$
QVina2 $\downarrow$	− 6.85$_{\pm 2.33}$	− 6.88$_{\pm 2.12}$	6.94$_{\pm 2.25}$
QVina2 (Top-10%) $\downarrow$	–	− 7.77$_{\pm 2.61}$	− 7.69$_{\pm 2.62}$
Lipinski $\uparrow$	3.35$_{\pm 1.14}$	**4.28**$_{\pm 1.19}$	**4.31**$_{\pm 1.18}$
SA $\uparrow$	0.72$_{\pm 0.13}$	0.68$_{\pm 0.14}$	**0.74**$_{\pm 0.14}$
QED $\uparrow$	0.47$_{\pm 0.20}$	0.47$_{\pm 0.22}$	0.46$_{\pm 0.23}$
logP	0.89$_{\pm 2.73}$	1.0$_{\pm 2.87}$	1.69$_{\pm 2.98}$
H-acceptors	6.08$_{\pm 3.86}$	5.98$_{\pm 3.92}$	5.83$_{\pm 3.88}$
H-donors	3.35$_{\pm 2.46}$	3.35$_{\pm 2.56}$	3.28$_{\pm 2.39}$
nAtoms	22.75$_{\pm 9.9}$	23.33$_{\pm 9.68}$	24.8$_{\pm 9.54}$

QuickVina2 is employed for docking. Mean values across all 100 targets with standard deviations are given as subscripts. QVina2 results are in units kcal/mol

**Table 2:** Pose and interaction performance on the CrossDocked2020 test set and ligands generated using DiffSBDD, TargetDiff and PoLiGenX

Metric	Test set	DiffSBDD	TargetDiff	PoLiGenX	PoLiGenX$^{\text {SA-guidance}}$
Vina Score $\downarrow$	− 5.49$_{\pm 2.82}$	− 1.21$_{\pm 6.29}$	− 5.08$_{\pm 2.42}$	− 5.04$_{\pm 2.67}$	− 5.10$_{\pm 2.63}$
Vina Score$^{\text {min}}$ $\downarrow$	− 5.99$_{\pm 2.49}$	− 4.25$_{\pm 3.32}$	− 6.11$_{\pm 2.24}$	− 5.85$_{\pm 2.32}$	− 5.87$_{\pm 2.38}$
Strain energy $\downarrow$	102.5$_{\pm 73.94}$	844.79$_{\pm 899.92}$	1044.26$_{\pm 1130.24}$	265.88$_{\pm 182.86}$	249.71$_{\pm 173.56}$
Clash count $\downarrow$	4.48$_{\pm 4.16}$	17.27$_{\pm 12.62}$	10.37$_{\pm 6.58}$	5.99$_{\pm 6.08}$	6.36$_{\pm 6.26}$
H-Bond recovery $\uparrow$	100	30.84$_{\pm 16.03}$	27.5$_\pm {15.44}$	47.94$_{\pm 24.68}$	50.12$_{\pm 26.71}$

Autodock Vina is employed for pose scoring and local energy minimization. Mean values across all 100 targets with standard deviations are given as subscripts. Vina scores and strain energies are in units kcal/mol. Vina score$^{min}$ denotes the Vina score after local minimization. H-Bond recovery is given in percentage

Table 1 summarizes the docking performance and chemical properties of ligands generated by PoLiGenX compared to ligands in the CrossDocked test set. Docking scores calculated using QuickVina2 indicate that PoLiGenX-generated ligands achieve an improved mean docking score of $-6.88 \pm 2.12$, with a notable improvement to $-7.77 \pm 2.61$ for the top 10% of generated samples. In comparison, the CrossDocked test set shows a mean docking score of $-6.85 \pm 2.33$. Furthermore, the ligands generated by PoLiGenX consistently adhere to Lipinski’s Rule of Five, suggesting good potential for therapeutic application, while mainting a high molecule validity of around 85%. These results collectively demonstrate that PoLiGenX produces ligands with therapeutic relevance, while simultaneously maintaining chemical diversity and favorable bioavailability characteristics.

Figure 5 visually compares ligands from the CrossDocked test set with four randomly selected ligands generated per target by PoLiGenX. Qualitative assessment confirms that PoLiGenX-generated ligands closely maintain the topological features of the reference ligands. Quantitatively, PoLiGenX achieves an average chemical similarity of 0.33 and shape similarity of 0.87, surpassing the performance of EQGAT-diff.

Synthetic accessibility (SA) of the ligands was evaluated using RDKit’s SA-score [31]. PoLiGenX-generated ligands exhibit a mean SA-score of 0.68, which is superior to the CrossDocked test set (mean SA-score of 0.72). Additionally, compared to other unconditional target-aware diffusion models such as DiffSBDD [5] and TargetDiff [2], each reporting a mean SA-score of 0.58 (as obtained from Le et al. [3], Table 5), PoLiGenX achieves improved synthetic accessibility when utilizing seed ligands as reference inputs.

Building upon recent advances by Cremer et al. [1], we integrate importance sampling guidance into the PoLiGenX model to bias diffusion trajectories toward ligands with improved synthetic accessibility (SA). For this purpose, we train the diffusion model along with an encoder network and a dedicated surrogate model. The surrogate model, branching from the diffusion network at its final layer, specifically predicts SA scores. Besides the SA-guidance, we incorporate a latent seed embedding *z*, achieving a mean SA score of 0.74 for the generated ligand set, as presented in Table 1.

We further assess the generated ligand poses and summarize our findings in Table 2. Initially, we calculate Vina scores for the raw ligand poses and subsequently apply local energy minimization (Vina score$^{\text {min}}$). The CrossDocked test set exhibits a mean Vina (pose) score of $-$5.49 kcal/mol, while the two sets generated by PoLiGenX achieve slightly lower mean Vina scores of $-$5.04 and $-$5.10 kcal/mol, respectively. In contrast, raw poses generated by DiffSBDD [5] display significantly less favorable conformations, evident from their poorer mean Vina score of $-$1.21 kcal/mol, accompanied by a high median strain energy of 844.79 kcal/mol and an average of 17.27 steric clashes. Even after applying local energy minimization, DiffSBDD-generated poses remain inferior to the unoptimized PoLiGenX poses.

Performance data for TargetDiff, extracted from Section 4.5 of Harris et al. [32], indicate a median strain energy of 1241.7 kcal/mol and an average of 9.08 steric clashes, further highlighting the superior performance of PoLiGenX. Using the code-base from TargetDiff, we were only able to evaluate 94 out of the 100 CrossDocked test targets. The results for TargetDiff on the 94 evaluated targets are shown in Table 2 as well. We observe that PoLiGenX also maintains lower strain energies and reduced number of steric clashes.

The two ligand sets generated by PoLiGenX, conditioned on latent embeddings and latent-SA embeddings, yield median strain energies of 265.88 kcal/mol and 249.71 kcal/mol, respectively. These results underscore the effectiveness of PoLiGenX in generating conformationally favorable ligand poses by leveraging seed ligand information to constrain diffusion trajectories. This conclusion is reinforced by comparable mean Vina scores following local energy minimization, as detailed in Table 2. In Fig. 6, we show two generated ligands from PoLiGenX in the binding pocket of PL-complex with PDB id 20gs. We observe that the generated poses are superior to the reference ligand regarding Vina score, while the second generated ligand also achieves a higher synthetic accessibility of 0.84 vs. 0.82.

Finally, we evaluate hydrogen bond interaction profiles using PoseCheck [32] with default settings. Binary interaction fingerprints, representing hydrogen bond donor/acceptor interactions between ligands and their respective pockets, were computed for 100 reference ligands as ground truth. Comparison with the corresponding hydrogen bond profiles for each generated ligand set reveals superior hydrogen bond recovery rates of 47.94% and 50.12% for PoLiGenX, significantly surpassing DiffSBDD’s 30.84% recovery rate as well as TargetDiff’s rate of 27.5%.

The inclusion of the importance sampling algorithm to bias the generation of ligands next to the latent-conditioning is an extension to the work published in [19], while our additional analysis including locally minimized ligand poses, clash count evaluation as well as hydrogen-bond interaction recovery advances previous work with further evidence of its usefulness.

**Fig. 7:**
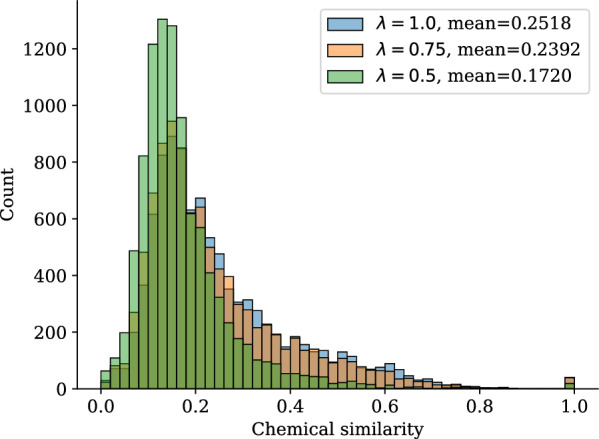
Density plot for chemical similarity of generated ligands from PoLiGenX with varying $\lambda$ control parameter. With increasing $\lambda$, the latent *z* of reference/seed ligand $M_0$ is preserved such that generated ligands exhibit higher chemical similarity to $M_0$

Finally, the controllability of PoLiGenX is enhanced by a scaling parameter $\lambda \in (0, 1]$ (see Eq. (2)), which adjusts the influence of the latent embedding *z*. When $\lambda$ approaches 0, PoLiGenX reverts to an unconditional EQGAT-diff model. By varying $\lambda$, we observe that higher values improve the chemical similarity of generated ligands to the reference, as shown in Fig. 7.

## Conclusions

We presented PoLiGenX, a diffusion-based model designed for controlled *de novo* ligand generation tailored specifically to protein binding pockets. PoLiGenX integrates latent embeddings derived from seed molecules, enabling the generation of ligands that satisfy both structural and chemical criteria relevant to their target protein sites while effectively preserving the shapes of reference ligands. This approach directly addresses a central challenge in drug discovery: achieving an optimal balance between specificity, diversity, and therapeutic viability.

By incorporating both chemical and shape information from seed ligands, PoLiGenX effectively supports applications in hit expansion and optimization. Moreover, the introduction of a tunable latent influence parameter allows researchers to flexibly navigate the trade-off between diversity and structural similarity according to specific requirements.

Evaluation results indicate that ligand poses generated by PoLiGenX achieve Vina scores comparable to those of seed ligands (without re-docking) and exhibit notably lower strain energies than ligands produced by unconditional generative diffusion methods like DiffSBDD. Additionally, PoLiGenX-generated ligands adhere consistently to established drug-likeness criteria, such as Lipinski’s Rule of Five.

Crucially, the model maintains substantial chemical diversity, enabling broad exploration of chemical space and increasing the potential for discovering novel therapeutic candidates with unique pharmacological profiles. We expanded the latent conditioning framework to encompass additional properties such as synthetic accessibility by employing an importance sampling algorithm proposed in [1], showing additional control can be achieved.

Future extensions of this work could involve accommodating dynamic protein conformations to facilitate ligand design for flexible binding sites. Further improvements may be realized by integrating advanced sampling techniques to more effectively uncover rare yet therapeutically promising chemical scaffolds.

## Data Availability

No datasets were generated or analysed during the current study.

## Derivation ELBO

We derive the variational lower bound for the latent diffusion model acting on data $x_0\in \mathbb {R}^D$, where the latent variable is lower-dimensional $z\in \mathbb {R}^K$ with $K <D$. Since we leverage diffusion models, which construct a sequence of latent variables of the same size, i.e., $x_1, x_2,..., x_T$ with $x_i\in \mathbb {R}^D$, the following general equation holds through marginalization

4 $$\begin{aligned} p(x_0)&= \int p(x_0, x_1, \dots , x_T)dx_1dx_2,\dots dx_T \end{aligned}$$

5 $$\begin{aligned}&= \int p(x_0, x_{1:T} ) dx_{1:T}. \end{aligned}$$

We use $p(x_{0:T})$ as abbreviation for $p(x_0, x_1, \dots x_T)$ and include two variational distributions (VD) $q(z|x_0)$ as well as $q(x_{1:T}|x_0)$. The first VD $q(z|x_0)$ is the commonly known from the VAE [33], while the second $q(x_{1:T}|x_0)$ is the Markov model [10] which factorizes as

6 $$\begin{aligned} q(x_{1:T}|x_0) = \prod _{t=1}^T q(x_t|x_{t-1}), \end{aligned}$$

by the Markov assumption $(*)$ where the next state only depends on the previous. Note that this also states for the reverse. We develop the ELBO as

7 $$\begin{aligned} p(x_0)&= \int p(x_{0:T}, z) dx_{1:T} dz \nonumber \\&= \int \frac{p(x_{0:T}, z) q(x_{1:T}|x_0) q(z|x_0)}{ q(x_{1:T}|x_0) q(z|x_0)}dx_{1:T} dz = \mathbb {E}_{q(x_{1:T}|x_0)q(z|x_0)}\left[ \frac{p(x_{0:T}|z)p(z)}{q(x_{1:T}|x_0)q(z|x_0)} \right] \nonumber \\&{\mathop {=}\limits ^{*}} \mathbb {E}_{q(x_{1:T}|x_0)q(z|x_0)}\left[ \frac{p(x_T|z)p(z) \prod _{t=1}^T p(x_{t-1}|x_t, z)}{q(z|x_0) \prod _{t=1}^T q(x_t|x_{t-1})} \right] \nonumber \\&\ge \mathbb {E}_{q(x_{1:T}|x_0)q(z|x_0)}\left[ \log \left( \frac{p(x_T|z)p(z) \prod _{t=1}^T p(x_{t-1}|x_t, z)}{q(z|x_0) \prod _{t=1}^T q(x_t|x_{t-1})}\right) \right] \nonumber \\&= \mathbb {E}_{q(x_{1:T}|x_0)q(z|x_0)}\left[ \log \left( \frac{p(x_T|z)p(z)p(x_0|x_1,z)}{q(z|x_0)q(x_1|x_0)} \prod _{t=2}^T\frac{ p(x_{t-1}|x_t, z)}{q(x_t|x_{t-1})}\right) \right] , \end{aligned}$$

where we condition on $x_0$ for the forward variational distribution $q(x_t|x_{t-1}) = q(x_t|x_{t-1}, x_0)$ because we require it later to obtain closed-form solution to regress on. Note that we applied Jensen’s inequality, bounding the likelihood. We have as a side computation (using Bayes’ theorem)

8 $$\begin{aligned} \prod _{t=2}^T q(x_t|x_{t-1}, x_0)&= \prod _{t=2}^T \frac{q(x_{t-1}|x_t, x_0) q(x_t|x_0)}{q(x_{t-1}|x_0)} = \prod _{t=2}^T q(x_{t-1}|x_t, x_0) \prod _{t=2}^T \frac{q(x_t|x_0)}{q(x_{t-1}|x_0)} \nonumber \\&= \prod _{t=2}^T q(x_{t-1}|x_t, x_0) \cdot \frac{q(x_T|x_0)}{q(x_1|x_0)}, \end{aligned}$$

where $\prod _{t=2}^T \frac{q(x_t|x_0)}{q(x_{t-1}|x_0)}$ is a telescoping series cancelling out intermediate products. We can insert (8) into the product series in (7) to obtain

9 $$\begin{aligned} p(x_0)&\ge \mathbb {E}_{q(x_{1:T}|x_0)q(z|x_0)}\left[ \log \left( \frac{p(x_T|z)p(z)p(x_0|x_1,z)}{q(z|x_0)q(x_1|x_0)} \frac{q(x_1|x_0)}{q(x_T|x_0)} \prod _{t=2}^T\frac{ p(x_{t-1}|x_t, z)}{q(x_{t-1}|x_t, x_0)}\right) \right] \nonumber \\&= \mathbb {E}_{q(x_{1:T}|x_0)q(z|x_0)}\left[ \log \left( \frac{p(x_T|z)p(z)p(x_0|x_1,z)}{q(z|x_0)q(x_T|x_0)} \prod _{t=2}^T\frac{ p(x_{t-1}|x_t, z)}{q(x_{t-1}|x_t, x_0)}\right) \right] \nonumber \\&= \mathbb {E}_{q(x_{1:T}|x_0)q(z|x_0)}\left[ \log p(x_0|x_1, z) + \log \frac{p(z)}{q(z|x_0)} + \log \frac{p(x_T|z)}{p(x_T|x_0)} + \sum _{t=2}^T \log \frac{p(x_{t-1}|x_t, z)}{q(x_{t-1}|x_t, x_0)} \right] . \end{aligned}$$

Next, we evaluate the expectation, which is the (multi-dimensional) integral over the variational distributions $q(x_{1:T}|x_0)q(z|x_0)$, i.e., integrating over the domains of $x_{1:T}$ and *z*, to obtain

10 $$\begin{aligned} p(x_0) \ge&\mathbb {E}_{q(x_1|x_0)q(z|x_0)}[\log p(x_0|x_1, z)] + \mathbb {E}_{q(z|x_0)}[\log \frac{p(z)}{q(z|x_0)}] + \mathbb {E}_{q(x_T|x_0)q(z|x_0)}[\log \frac{p(x_T|z)}{q(x_T|x_0)}] \nonumber \\&+ \mathbb {E}_{q(x_{t-1}|x_t, x_0)q(x_{t}|x_0)q(z|x_0)}[\sum _{t=2}^T \log \frac{p(x_{t-1}|x_t, z)}{q(x_{t-1}|x_t, x_0)}], \end{aligned}$$

where the (reverse) KL divergence between distributions *q*(*x*) and *p*(*x*) reads

11 $$\begin{aligned} D_{KL}(q(x)||p(x)) = \mathbb {E}_{q(x)} [\log \frac{p(x)}{q(x)}]. \end{aligned}$$

Plugging Eq. (11) into (10), we obtain

12 $$\begin{aligned} p(x_0) \ge&\mathbb {E}_{q(x_1|x_0)q(z|x_0)}[\log p(x_0|x_1, z)] + D_{KL}(p(z)||q(z|x_0)) + \mathbb {E}_{q(x_T|x_0)q(z|x_0)}[\log \frac{p(x_T|z)}{q(x_T|x_0)}] \end{aligned}$$

13 $$\begin{aligned}&+ \mathbb {E}_{q(x_{t}|x_0)q(z|x_0)}[\sum _{t=2}^T D_{KL}(p(x_{t-1}|x_t, z)|| q(x_{t-1}|x_t, x_0))]. \end{aligned}$$

The first expectation in the ELBO is commonly known as the reconstruction loss, while the second expectation is defined as KL-prior regularization loss. For the third expectation, we make the assumption that the ambient space prior $x_T$ is independet of the latent variable *z*, i.e., $p(x_T|z)=p(x_T)$. It could be interesting to further investigate if the ambient prior can be influenced through a latent variable but due to simplicity, we remove it. This leads to simplyfing the third expectation into $D_{KL}(p(x_T) || q(x_T|x_0)$, where by design of the diffusion model and its noise-schedule, $q(x_T|x_0)$ should converge to a multivariate standard normal distribution *N*(0, *I*). The last expectation is the summation over $(T-1)$ KL-divergences to train the generative diffusion model matching the reverse $p(x_{t-1}|x_{t}, z)$ with the tractable $q(x_{t-1}|x_t, x_0)$. Instead of summing over all noise-levels indexed from $t=2$ until $t=T$ optimizing the derived ELBO, we only optimize $D_{KL}(p(z)||q(z|x_0))$ using the Maximum-Mean-Discrepancy (MMD) [25] loss instead of KL and uniformly sample a time-step from $t\sim U(2,T)$ to minimize $D_{KL}(p(x_{t-1}|x_t, z)|| q(x_{t-1}|x_t, x_0))]$.

## Code availability

The processed CrossDocked2020 dataset can be downloaded from https://github.com/pengxingang/Pocket2Mol/tree/main/data. The code for this study can be found on https://github.com/pfizer-opensource/e3moldiffusion/tree/poligenX.
